# The physiological basis of leader-follower roles in the dyadic alternating tapping task

**DOI:** 10.3389/fpsyg.2023.1232016

**Published:** 2023-11-30

**Authors:** Kenta Tomyta, Natsuki Saito, Hideki Ohira

**Affiliations:** ^1^Department of Cognitive and Psychological Sciences, Nagoya University, Nagoya, Aichi, Japan; ^2^Japan Society for the Promotion of Science, Tokyo, Japan; ^3^Graduate School of Informatics, Nagoya University, Nagoya, Aichi, Japan

**Keywords:** tapping, rhythm, EEG, communication, mirror neurons

## Abstract

**Introduction:**

Cooperative and collaborative behaviors are important concepts for co-creative communication. One of the key elements for these behaviors is the leader-follower roles in human communication. Leaders are those who maintain their own pace and rhythm, on the contrary, followers are those who follow the pace and rhythm of the other. Well-coordinated leader-follower roles would produce better cooperative and collaborative behaviors, which could promote co-creative communication.

**Methods:**

Here, to explore the physiological basis for the leader-follower roles, we conducted the dyadic alternating tapping task with electrocardiographic and electroencephalographic recordings. The task would be stable for modeling human communication in the laboratory because it includes timing control in tens of milliseconds and turn-taking. Given that human communications are complex and constantly fluctuating, this study estimated the degree of leader-follower with the state-space model. This model allowed us to calculate two parameters independently for estimating the degree of leader-follower of each participant: αSelf (degree of one’s tap(n) was explained by one’s tap(n-1)) and αPair (degree of one’s tap(n) was explained by one’s tap (n-1) and pair’s tap (n-1)).

**Results:**

The result showed heart rate synchronization in the group in which both participants had high αPair. Also, the high-frequency component of heart rate variability was positively correlated with αPair. EEG analyses suggested the deactivation of the mirror neuron system (increasing φ1) in the participants with higher αSelf than lower ones. The activation of the mirror neuron system (increasing φ2) was shown in the participants with lower αPair than higher ones.

**Discussion:**

These data of physiological basis for leader-follower roles could be useful for the constructivist approach to co-creative communication.

## Introduction

Rhythm plays a very important role in our social interaction. For instance, when two people work together to lift heavy objects, they need to synchronize their lifting timing. In conversation, rhythmic switching of speakers such as turn-taking is one of the most important elements (e.g., Pouw and Holler, 2022). The dyadic alternating tapping task is often used to investigate the psychological and neural basis of turn-taking (e.g., Kawasaki et al., 2017; Takamizawa and Kawasaki, 2019; Kurihara et al., 2022). In this task, two subjects were required to perform the rhythmic tapping alternately (anti-phase). This task could be used as a minimal experimental task to investigate rhythmical social interaction between two persons. Unlike actual conversational behavior, the dyadic alternating tapping task has the advantage of eliminating as much as possible factors other than rhythm, such as the content of the conversation.

In social interaction tasks with two participants, two types of role assignments occur in pairs: the “leader” who keeps his or her own pace and the “follower” who follows the pace of the pair (Konvalinka et al., 2010; Jiang et al., 2015; Takamizawa and Kawasaki, 2019). Marsh et al. (2009) suggested task performance in the leader-follower role enhancement. These previous studies in this research area suggested that the leader/follower role is a very important element in communication. Also, leader/follower roles were also observed in the dyadic alternating tapping task (Takamizawa and Kawasaki, 2019). However, the psychological and neural basis of the leader/follower role in the dyadic alternating tapping task, which deal with timing gaps ranging from tens to hundreds of milliseconds, remains unclear. Therefore, this study focused on physiological states such as electrocardiogram (ECG) and electroencephalogram (EEG) during the dyadic alternating tapping task to clarify the psychological and neural characteristics of the leader/follower role. Because the leader/follower roles are one of the important features in social interaction, the exploratory study could be useful for a constructivist approach to co-creative communication.

Many studies have reported that heartbeat synchrony occurs when two parties are interacting successfully in social interaction tasks (e.g., Kodama et al., 2018; McAfee, 2018; Balconi et al., 2019a,b). Thus, heartbeat synchrony could be useful as an indicator of social interaction between two parties. However, no studies have examined the relationship between leader/follower and heartbeat synchrony during dyadic alternating tapping tasks. We hypothesize that heartbeat synchrony occurs in leader-follower pairs. On the other hand, heart rate synchrony might not occur when the pair was a leader/leader pair.

We also focused on heartrate variability(HRV). The HRV could be classified into very-low frequency (VLF; 0.0033–0.04 Hz), low frequency (LF, 0.04–0.15 Hz), and high frequency (HF, 0.15–0.4 Hz). The VLF-HRV reflects long-term regulation mechanisms, thermoregulation, and hormonal mechanisms (Laborde et al., 2017). The LF-HRV reflects a mix between sympathetic and parasympathetic tones (Laborde et al., 2017), and the HF-HRV reflects a parasympathetic tone (Reyes del Paso et al., 2013). Additionally, the LF/HF ratio has been used as an index of sympathovagal balance (Forte et al., 2019). Previous studies reported higher HRV was correlated with a higher cognitive function such as memory and attention (e.g., Frewen et al., 2013; Williams et al., 2016). Moreover, previous studies suggested a higher HRV was associated with emotion and communication (Porges, 2003; Porges, 2007; Smith et al., 2020). Thus, we could hypothesize that participants with higher HRV intended to be leaders in the dyadic alternating tapping task.

For analyses of EEG in the dyadic alternating tapping task, we focused on the Phi complex. The Phi complex is suggested neural indices of social interaction (Tognoli et al., 2007). Phi complexes are classified as Phi1 (approximately 10–12 Hz) and Phi2 (approximately 12–13 Hz) (Tognoli et al., 2007). The Phi complex could be associated with mirror neuron which activated social interaction (e.g., Decety and Lamm, 2007; Molenberghs et al., 2009). The mirror neuron which consists of the inferior frontal gyrus and the inferior parietal was activated in social interaction (Rizzolatti and Craighero, 2004). Hirao and Masaki (2021) investigated the relationship between the Phi complex and leader/follower in an in-phase synchronization task. The study showed no correlations between the Phi complex and leader/follower. In other words, they suggested the mirror neuron system was not involved with the leader-follower role. However, whether the mirror neuron system is involved with the leader-follower role in anti-phase is not clear. The psychological and neural mechanisms of the in-phase interactions might differ from that of anti-phase interactions. Therefore, this study investigated the relationship between the Phi complex and leader/follower in anti-phase interaction (the dyadic alternating tapping task). We hypothesized that the leader showed higher Phi1 and the follower showed higher Phi2.

## Methods

### Participants

This study recruited 82 participants (41 pairs, 42 men and 40 women, Mage 20.33 years). One of the pairs was excluded from the analysis due to discrepancies in understanding the teaching of the experiment. This study was approved by the Ethics Committee of Nagoya University and conducted in accordance with the relevant guidelines (approval number NUPSY-180914-G-02).

### Task (resting ECG recording and the dyadic alternating tapping task)

**Figure 1:**
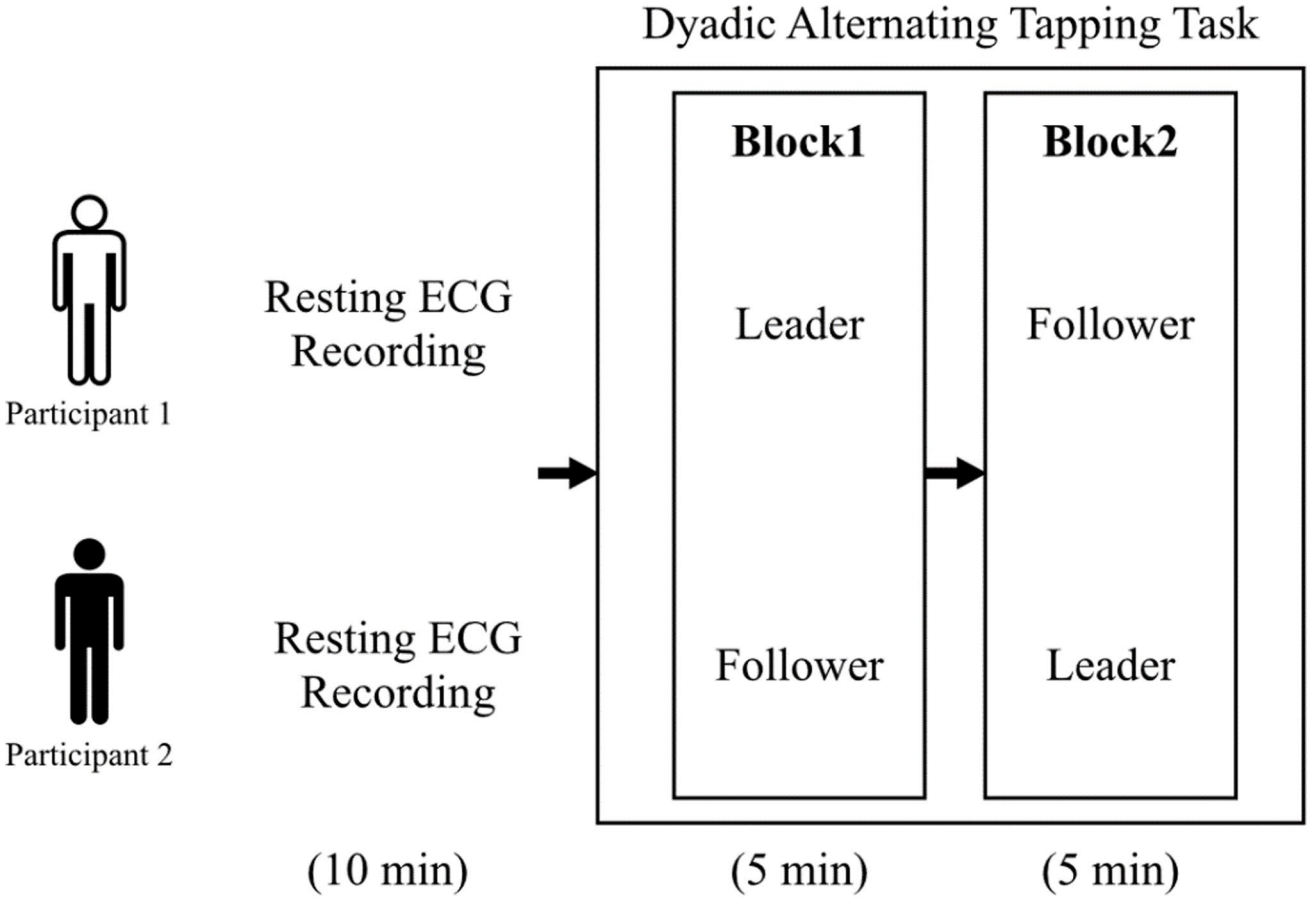
Schematic diagram of the experimental procedure.

First, a 10-min resting ECG was recorded. Then, the dyadic alternating tapping task was conducted. In this task, participants were required to alternate rhythmically press the keyboard with PC in pairs. Participants were seated side-by-side across a partition and performed the task in two blocks. In Block 1, one participant was assigned to a leader who was allowed to change the rhythm, the other was assigned to a follower who was instructed to keep the tapping pace of the leader for 5 min. In Block 2, the participant who was the leader in Block 1 was assigned to the follower for 5 min, and vice versa. During the task, ECG and EEG were recorded. Their tapping timings were recorded with Psychopy v1.80.30 (Peirce, 2009) (Figure 1).

### Tapping data processing

We calculated two types of tapping interval; Individual tapping interval (Individual tapping interval A (n) = tapping onset A (n + 1) − tapping onset A (n))and Pairs tapping interval (Pairs tapping interval A and B (n) = tapping onset A (n) − tapping onset B (n)).

### Estimation of leader/follower

To quantify the degree of leader/follower in the dyadic alternating tapping task, a state-space model was used (See Supplementary material for the model details). The model has αSelf and αPair parameters. αSelf was the degree of tapping at a certain point in time explained by the tapping interval of the previous self and αPair was the degree explained by the tapping interval between the previous partner and oneself. Therefore, αSelf was assumed to be the degree of leader-like tapping at one’s own pace and αPair was assumed to be the degree of follower-like tapping at a pace considering others. αPair was assumed to be the degree of follower-like tapping at a pace that takes others into account.

### ECG recording and processing

An ECG was recorded at 1,000 Hz with a BIOPAC MP 150 device (BIOPAC Systems Inc., Goleta, CA, United States) and AcqKnowledge software (BIOPAC Systems Inc., Goleta, CA, United States). The R-R intervals were extracted from 10-min resting ECG data with the AcqKnowledge software. To estimate heartbeat synchronization between pairs, the R-R intervals correlation coefficient was calculated. The R-R intervals were also imported into Kubios HRV Standard software (Version 3.5.0; Niskanen et al., 2004) to obtain power spectrum density values. We conducted the fast Fourier transform for the R-R interval to calculate VLF, LF, and HF power of the HRV.

### EEG recording and processing

EEGs were recorded using the Emotive EPOC (14 channels, 10–20 system, impedance <10 kΩ) and converted to an average reference after 0.1–40-Hz band-pass filtering. Kotowski et al. (2018) reported the validation of the Emotiv EPOC. In offline analyses, artifacts were removed with an automatic independent component analysis provided by MNE-Python (Gramfort et al., 2013). Moreover, epoching was performed in 1-s segments. Epochs that exceeded ±200 μV were excluded from further analyses. Then, we performed frequency analyses for the EEG data to calculate Phi1(10-12 Hz) and Phi2(12-13 Hz). We analyzed the difference in Phi1 and Phi2 amplitude between the left and the right hemisphere as in previous studies. Previous studies analyzed the Phi complex per electrode. Considering the effects of artifacts, error of signal sources estimation, and so on, however, this study calculated the Phi complex from the average power of all electrodes in the right and left hemispheres.

### Data analyses

We conducted correlation analyses between HRV and aPair/αSelf with Bonferroni correction. For the analyses of heartbeat synchronization, the correlation coefficient of the R-R intervals was calculated. Then, experimental pairs were divided into three groups (both αPair values higher than the median; one αPair was higher than the median, and both αPair values lower than the median). The correlation coefficients of R-R intervals for these three groups were analyzed by analysis of variance with Bonferroni correction.

For the EEG analyses, the subjects were divided between the low and high groups by the median of αPair and αSelf, respectively. The subjects were also divided between the low and high groups by the median fold of αPair and αSelf, respectively. The magnitudes of Phi1 and Phi2 were compared between the groups with Wilcoxon rank sum test with Bonferroni correction (Figure 1).

## Results

### Heartbeat synchronization

**Figure 2:**
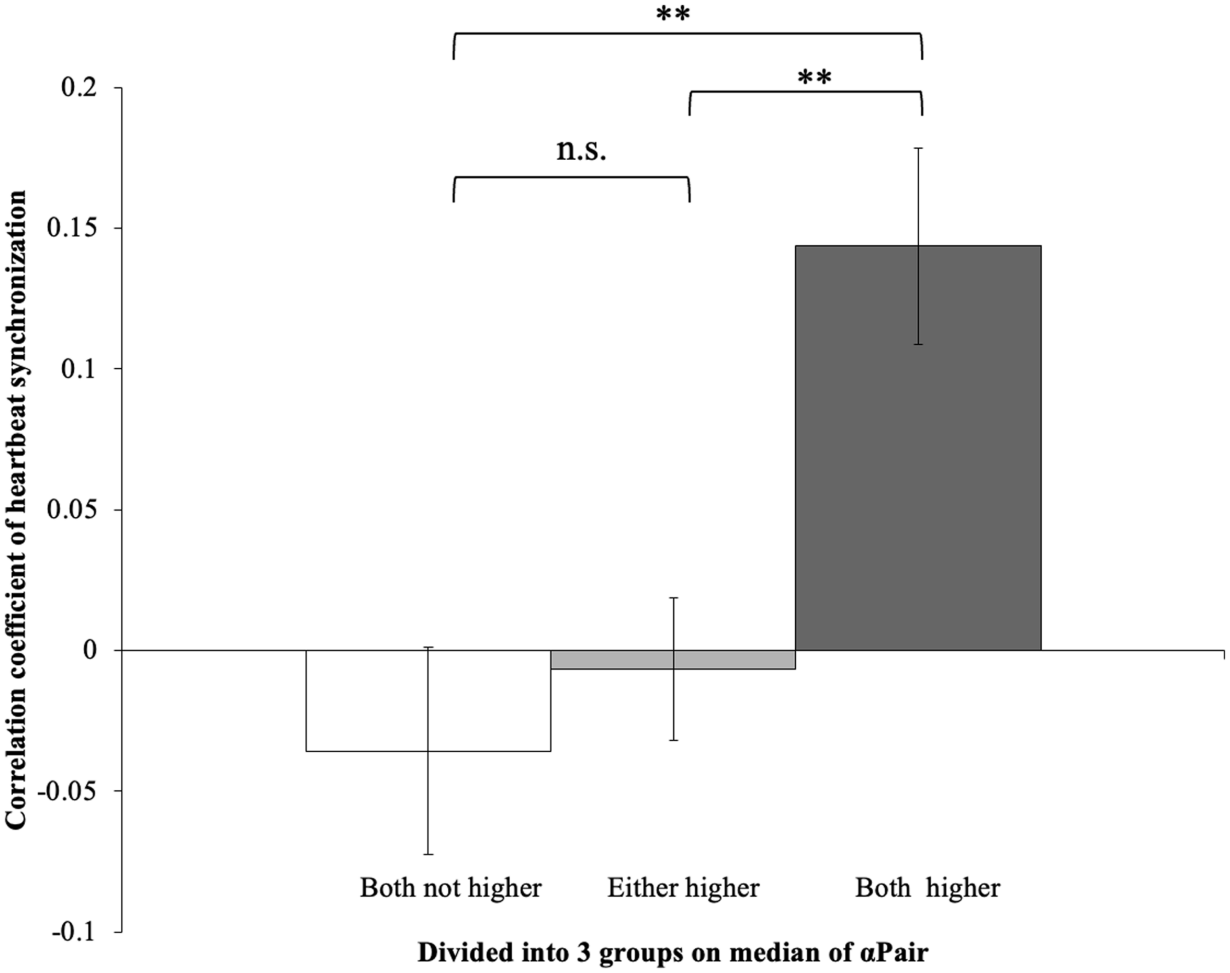
Correlation coefficient of heartbeat synchronization in Block1. The pairs in which both had higher αair showed higher heartbeat synchronization than pairs in which only one person had a higher αPair or both participants had lower values (*t*_(35)_ = −3.54, *p* < 0.01; *t*_(35)_ = −3.49, *p* < 0.01).

**Figure 3:**
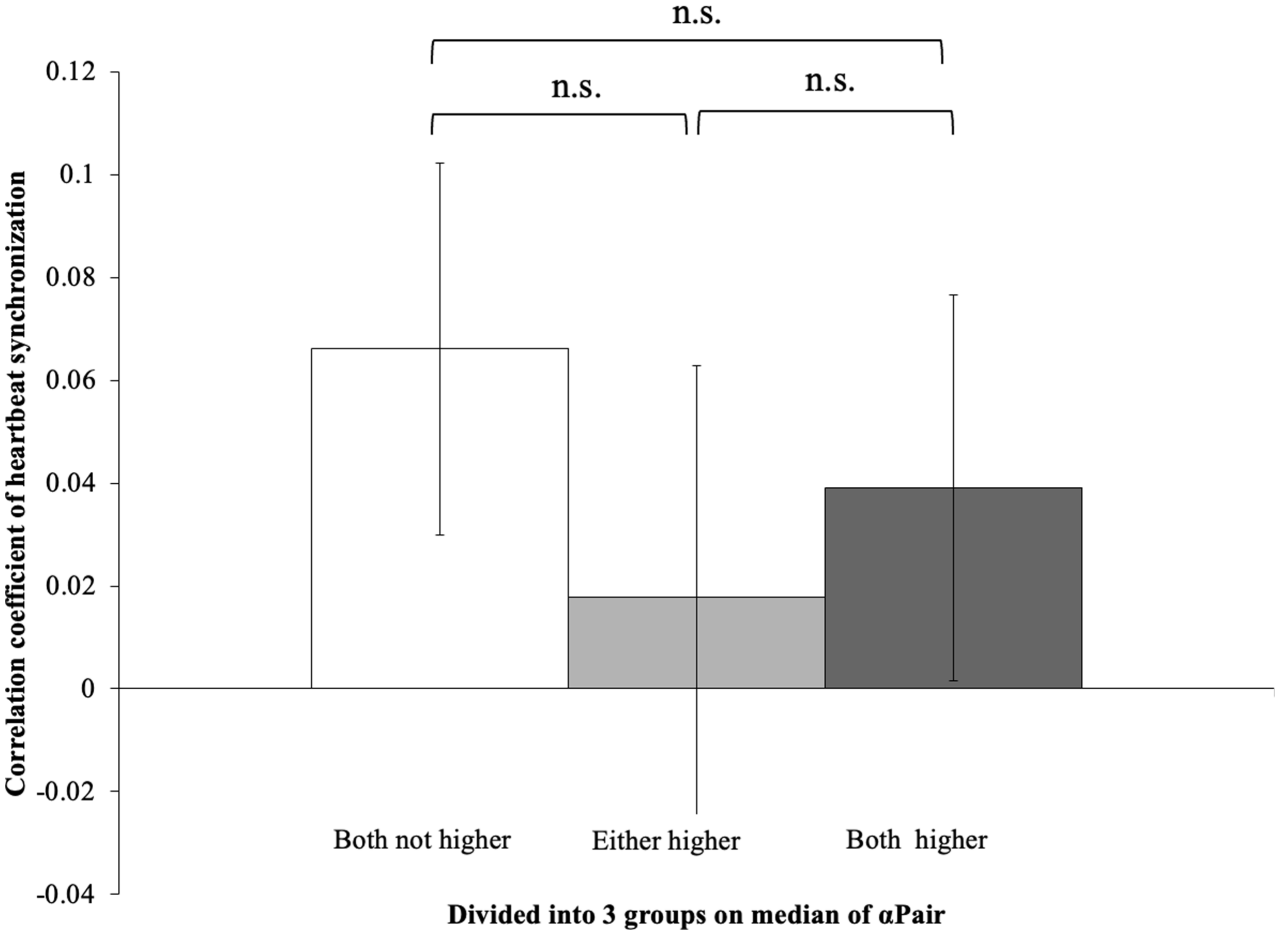
Correlation coefficient of heartbeat synchronization in Block1. No differences were shown in each block (*F*_(2, 35)_ = 0.37, *p* = 0.70).

In the Block1, the pairs in which both had higher αair showed higher heartbeat synchronization (R-R intervals correlation coefficient) than pairs in which only one person had a higher αPair or both participants had lower values (*t*_(35)_ = −3.54, *p* < 0.01; *t*_(35)_ = −3.49, *p* < 0.01) (Figure 2). Meanwhile, there was no difference between pairs where only one person had a higher αPair and pairs where both participants had lower αPair group (*t*_(35)_ = −3.54, *p* = 0.518). Moreover, in Block2, no significant differences were shown in all groups (*F*_(2, 35)_ = 0.37, *p* = 0.70) (Figure 3).

### Heartrate variability

A positive correlation was found between αPair and HF in both Block 1 (*r* = 0.23, *p* < 0.05). However, there was no correlation between αPair and HF in both Block 2 (*r* = 1.4, *p* = 0.96). Also, there were no correlations between αPair and the LF or LF/HF ration in both Block 1 and 2 (*r* = 1.82, *p* < 0.725; *r* = 1.42, *p* < 0.834). There were no correlations between αSelf and the LF, HF or LF/HF ration in both Block 1 and 2 (Block1: *r* = 0.08., *p* = 0.81; *r* = −0.04., *p* = 0.12; *r* = 0.04., *p* = 0.88: Block2: *r* = −0.07, *p* = 0.85; *r* = −0.08., *p* = 0.93; *r* = 0.03., *p* = 0.77).

### EEG

**Figure 4:**
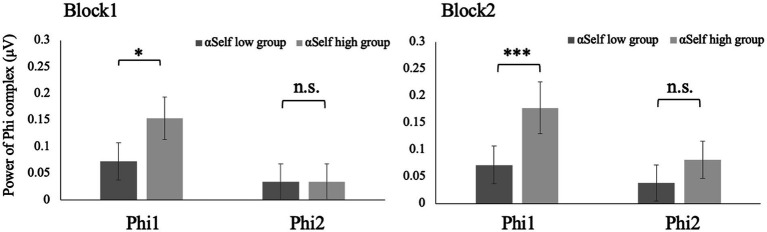
Phi1 of the participants with higher αSelf was larger than those with lower αSelf in both Block 1 and 2 (*w* = 276, *p* < 0.05; *w* = 122, *p* < 0.001).

**Figure 5:**
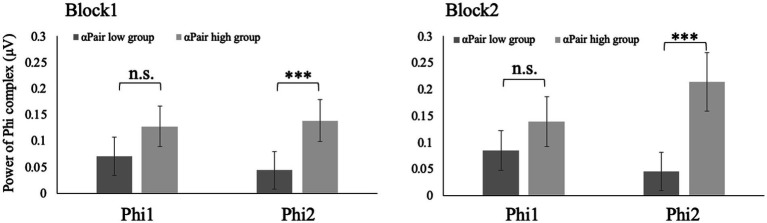
Phi2 of the participants with higher αPair was larger than that with lower αPair in both Block 1 and 2 (*w* = 737, *p* < 0.001; *w* = 753, *p* < 0.001).

Phi1 of the participants with higher αSelf was larger than those with lower αSelf in both Block 1 and 2 (*w* = 276, *p* < 0.05; *w* = 122, *p* < 0.001) (Figure 4). No significant differences in the Phi1 were observed between large and small αPair in both Block 1 and 2 (*w* = 543, *p* = 0.11; *w* = 482, *p* = 0.68). Phi2 of the participants with higher αPair was larger than that with lower αPair in both Block 1 and 2 (w = 737, *p* < 0.001; w = 753, *p* < 0.001) (Figure 5). No significant differences in the Phi2 were observed between large and small αSelf in both Block 1 and 2 (*w* = 482, *p* = 0.34; *w* = 289, *p* = 0.08).

## Discussion

This study aimed to explore the physiological basis for the leader-follower roles in communicative situations. For this purpose, we conducted the dyadic alternating tapping task with electrocardiographic and electroencephalographic recordings. The degree of adaptation to the partner was estimated using a state-space model which has the parameters αSelf and αPair. We investigated the relationship between these parameters and ECG/EEG data.

The pairs in which both participants had higher aPair values showed greater heartbeat synchronization compared to pairs where only one person had a higher value or both participants had lower in Block1. Previous studies showed that heartbeat synchronization occurs during interpersonal interactions. Marsh et al. (2009) suggested that task performance is higher when heartbeat synchronization occurs than when not. Our results suggested that heartbeat synchronization occurred when both participants had the intention to synchronize with each other. However, no significant differences were shown in Block 2. The discrepancy between the results of Block 1 and Block 2 is difficult to interpret. The most likely order effect is considered. In this study, subjects who were followers in Block 2 had previously experienced being leaders in Block 1. Therefore, further research would need to experiment with a between-subjects design rather than a within-subjects design.

We hypothesized that participants with higher HRV showed higher αPair. Consistent with our hypothesis, HF-HRV was positively correlated with αPair in Block1. This result suggests that when participants with higher HRV may take on the leader role in the dyadic alternating tapping task, they can keep pace with the partner to facilitate this task. Thus, this result was consistent with previous studies that a higher HRV was associated with emotion and communication. In addition, the HF-HRV reflects parasympathetic tone (Reyes del Paso et al., 2013). Therefore, the results suggested the more tapping was done considering the overall pace, the more parasympathetic dominance was indicated.

Hirao and Masaki (2021) showed no correlation between leader/follower role and Phi complex in in-phase synchronization tasks. Meanwhile, this study showed that Phi1 of the participants with higher αSelf was larger than that with lower αSelf in both Block 1 and 2. The power of Phi1 indicated inhibition of mirror neurons (Decety and Lamm, 2007; Tognoli et al., 2007; Molenberghs et al., 2009). Thus, when participants intended to keep their pace, mirror neuron was more deactivated. Phi2 of the participants with lower αPair was larger than that with higher αPair in both Block 1 and 2. The power of Phi2 indicated the activation of mirror neurons. Thus, when participants intended to keep the pace of the pair, the mirror neuron was more activated. These results suggested Phi complex or mirror neuron could be associated with anti-phase but not in-phase communication. The limitation of this study was that we used Low-cost EEG (Emotive EPOC with 14 channels). Our results suggested that Phi complex could be detected with Low-cost EEG. This report makes a significant contribution to EEG measurements in communication research. Kotowski et al. (2018) reported the validation of the Emotiv EPOC. However, further study needs to confirm that the results could be reproduced using EEG with 32 or more channels.

Finally, we discuss the social applications of our research. In everyday life, people engage in cooperative and competitive communication (e.g., Miyake, 2010). Previous studies have emphasized the significance of leaders and followers in communication dynamics (e.g., Hirao and Masaki, 2021). Our results showed that there is a physiological basis for leader-follower roles, which can have important implications for various real-world scenarios, such as forming partnerships in business. Furthermore, our findings will be useful in helping the effective integration of robots into social interactions. In order for people to communicate with robots in a way that they feel comfortable, considering the concept of leader-follower dynamics in robot design parameters becomes crucial.

In conclusion, this study aimed to investigate the psycho-neuro basis of leader-follower roles in the dyadic alternating tapping task. For this purpose, we estimated the degree of leader-follower to set αPari & αSelf parameters with the state-space model. As a result, αPari & αSelf parameters were correlated with ECG and EEG. The leader/follower roles were one of the key elements for human communication (Konvalinka et al., 2010; Jiang et al., 2015; Takamizawa and Kawasaki, 2019). Therefore, these results could be useful for the constructivist approach to co-creative communication.

## Data availability statement

The raw data supporting the conclusions of this article will be made available by the authors, without undue reservation.

## Ethics statement

The studies involving humans were approved by the Ethics Committee of Nagoya University. The studies were conducted in accordance with the local legislation and institutional requirements. The participants provided their written informed consent to participate in this study.

## Author contributions

KT worked on EEG and wrote this manuscript. NS worked on Tapping data and ECG. HO designed the experiment. All authors contributed to the article and approved the submitted version.

## Funding

This work was supported by grants from the Japan Society for the Promotion of Science (JSPS) (17942715, 21375683) and Japan Science and Technology Agency (JST) (JPMJCR21P1).
