# Supplementary material for: The physiological basis of leader-follower roles in the dyadic alternating tapping task
Source: Front Psychol. 2023 Nov 30;14:1232016. doi: 10.3389/fpsyg.2023.1232016 (PMC10720364; doi:10.3389/fpsyg.2023.1232016)
Supplement: Supplementary file 1 [file Table_1.DOCX]

**Model**

A state-space model was employed to account for the temporal changes in the observed data. This model aimed to estimate parameters that represent the participants' tapping behavior characteristics based on the observed tapping interval data. Specifically, the regression coefficients for the participant's own previous tapping interval and the tapping interval of their partner in the previous trial were estimated with respect to the participant's mean tapping interval. These coefficients were used to estimate parameters (αSelf) representing how the participant's previous tapping interval affects their next tapping interval and (αPair) representing the interaction between participants.

A state-space model was used with the tapping interval data of a participant as the observed data (y) and the tapping interval from one participant's tapping to the other participant's tapping (b) as the observed data. The model is given by a state equation and an observation equation.

State equation:

if t=1, the state variable mu_t is initialized as:

mu_t = f(muZero, W_t)

if t≠1, mu_t is updated as follows:

mu_t = f(αSelf * mu[t-1] + αPair * b[t] + β, W_t)

Here, mu represents the participant's mean tapping interval at each tapping, and muZero is the initial state of the mean interval. The parameter αSelf represents how the participant's own previous tapping interval at time t-1 affects their current tapping interval at time t, while αPair is the regression coefficient of the current tapping interval with respect to the tapping intervals of the participant and their partner in the previous trial. β is the constant term of mu_t at time t.

Observation equation:

The observed tapping interval data of a participant is denoted as y_t, and is modeled as a function of the state variable mu_t and the variance of tapping intervals V_t:

y_t = g(mu[t], V_t)

The state and observation variables are assumed to follow normal distributions with means of 0 and variances of W and V, respectively.
